# Niobium Phosphorus Trichalcogenide (NbPS_3_): A Promising Monolayer Material for Magnetic and Optoelectronic Applications

**DOI:** 10.1021/acsomega.5c07942

**Published:** 2025-12-19

**Authors:** Leonardo de Souza Barbosa, Willian Oliveira Santos, David Lima Azevedo, Edvan Moreira

**Affiliations:** † Postgraduate program in Aerospace Engineering, State University of Maranhão (UEMA), Cidade Universitária Paulo VI, 65055-310 São Luís, Maranhão, Brazil; ‡ Postgraduate program in Physics, Federal University of Sergipe (UFS), Cidade Univ. Prof. José Aloísio de Campos, 49107-230 São Cristóvão, Sergipe, Brazil; § Institute of Physics, University of Brasilia (UnB), Campus Universitário Darcy Ribeiro - Asa Norte, 70919-970 Brasília, Distrito Federal, Brazil; ∥ Department of Physics, State University of Maranhão (UEMA), Cidade Universitária Paulo VI, 65055-310 São Luís, Maranhão, Brazil

## Abstract

In this study, a novel monolayer of niobium phosphorus trichalcogenide (NbPS_3_) is investigated. The structural, electronic, magnetic, optical, vibrational (phonons, infrared (IR), and Raman), and thermodynamic properties were studied by using density functional theory. Electronic and magnetic properties showed that NbPS_3_ is a spin-dependent half-metallic with potential for spintronic application devices. NbPS_3_ absorbs in the visible region, corresponding to the red color (∼640 to 660 nm), and is sensitive to the plane of polarization of the incident light. Furthermore, the phonon dispersion calculation shows only positive frequencies, indicating dynamic stability, and the theoretical peaks of the IR and Raman spectra were studied and assigned, providing a solid basis for comparison with theoretical and experimental data. Ab initio molecular dynamics simulations showed the thermal stability of NbPS_3_ at temperatures of 300 and 600 K. In addition, thermodynamic properties were calculated and discussed. The results demonstrate that the NbPS_3_ monolayer is feasible, with potential applications in optoelectronics and spintronics.

## Graphical abstract

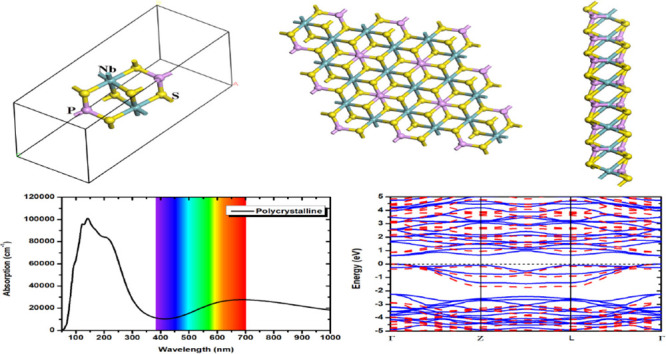

## Introduction

The advances in material characterization have opened novel technological applications in conductors, semiconductors, optoelectronics, spintronics, etc. Consequently, studies in graphene and its lack of an electronic bandgap^1^ were one of the major contributors to the development of two-dimensional (2D) structures, such as transition metal dichalcogenides (TMDs),^2–4^ transition metal dihalides (TMDHs),^5,6^ MXenes,^7^ and others. TMDs are semiconductors with the general formula MX_2_, where M denotes a transition metal and X denotes a chalcogen, and the most well-known TMDs are MoS_2_ and WS_2_. TMDs display direct bandgap, semiconductor features, strong spin–orbit coupling, and favorable mechanical properties, which make them suitable for applications, e.g., in spintronics, optoelectronics, and energy harvesting.^2,3^ TMDHs are another class of 2D materials that have been investigated recently due to their magnetic properties. TMDH materials are typically described by the formula MX_2_, where M is a transition metal and X denotes a halogen (F, Cl, and I).^5,6,8^ TiBr_2_-2H monolayer is an example of TMDH with promising optical and excitonic characteristics, positioning it as a potential material for optoelectronic and valleytronic applications.^9^ Other TMDHs have been studied for applications in, e.g., information storage devices and spintronic technologies.^10–15^ MXenes are another class of 2D materials that have recently been extensively investigated, having the chemical formula M_*n*+1_X_*n*_T_*x*_, where X can be N or C, and T is the termination groups on the surface (−F, −OH, −Cl, −O, Br), where *x* is the number of terminations.^7,16–18^ MXenes exhibit remarkable conductivity and abundant surface functionalities, making them suitable for a variety of uses and applications, including supercapacitor development,^7^ energy storage, and energy conversion applications.^18–20^ The properties and applications of all of these materials showed the importance of theoretical and experimental investigations of novel monolayer classes.

The class of two-dimensional transition-metal phosphorus trichalcogenides with chemical formula MPX_3_ is a layered material that preferably crystallizes in two space groups, *C*2/*m* (No. 12) and R3 (No. 148).^21^ Generally, the MPX_3_ monolayer consists of metal transition atoms organized in a honeycomb lattice. Each hexagon is centered on the P–P dimer, vertical to the layer.^21^ MPX_3_ materials are usually semiconducting materials whose bandgap varies between 1.2 and 3.5 eV.^21–24^ These wide bandgaps are responsible for the enhanced light absorption efficiency compared to other 2D materials, such as TMD.^24,25^ Transition-metal phosphorus trichalcogenides are peculiar due to several reasons, including tunable low-dimensional magnetism, low-dimensional ferroelectricity, and possible applications in Li-ion batteries and hydrogen storage.^21^ Beyond that, ferroelectric phenomena are used, for example, to employ MPX_3_ materials in the manufacture of data device storage and fire sensors.^24^ MPX_3_ materials present magnetic properties, and most experimental studies on magnetism in MPX_3_ are restricted to bulk crystals. Most investigations of magnetic phenomena in MPX_3_ monolayers are theoretical; however, FePS_3_ is supported by experimental evidence.^24^ The magnetic properties of MPX_3_ materials (bulk and monolayer) are extremely important for spintronic applications, which could lead to novel devices with potential applications. Furthermore, DFT calculations reported interesting optical properties for various MPX_3_ single layers with absorption in visible light.^24,25^ In addition, photoluminescence was observed for many MPX_3_ compounds.^24,26^ MPX_3_ materials are reported to have advanced applications, such as catalysis (photocatalysis and electrocatalysis), field effect transistors, photodetectors, chemical sensors, and energy storage devices.^21,24^ All of these properties and applications of two-dimensional transition-metal phosphorus trichalcogenide materials encourage the search for new structures and the study of their properties, which offer a wide range of characteristics and applications.

The use of niobium (Nb) has increased in numerous fields, including microalloyed steels, superalloys, thin-film technologies, medical implants, superconducting materials, and capacitors.^18,27^ Thus, because of the properties and applications of niobium, it is natural to study novel niobium-based monolayers.

To date, niobium-based MPX_3_ monolayers have not been reported in the literature. This investigation provides the first prediction of NbPS_3_, revealing interesting properties and positioning NbPS_3_ as a promising new 2D structure for future spintronic and optoelectronic applications. In this paper, a novel monolayer of phosphorus trichalcogenides (NbPS_3_) with a niobium transition metal was investigated. Electronic, magnetic, optical, and vibrational properties (including phonon dispersion, IR, and Raman), thermodynamic properties, and quantum dynamics were studied to describe the features of NbPS_3_.

The paper is organized as follows: In the Methodology and Computational Details section, all computational methods employed to obtain the properties of the NbPS_3_ monolayer are presented. In the Results and Discussion section, all results found for the physical properties of the NbPS_3_ monolayer are presented and discussed. Finally, in the Conclusions section, the key findings from the results are presented, summarizing the main results.

## Methodology and Computational Details

The computational simulations were performed using the CASTEP code (Cambridge Sequential Total Energy Package),^28,29^ following a methodology similar to previous work.^30^ CASTEP software is based on density functional theory (DFT)^31,32^ to calculate, for example, electronic, magnetic, optical, thermodynamic, and vibrational properties of periodic structures. The Perdew–Burke–Ernzerhof (PBE) functional^33^ of the generalized gradient approximation (GGA) was used to evaluate all the properties of the NbPS_3_ monolayer. A HSE06 hybrid functional^34,35^ single-point energy calculation was performed starting from the GGA-PBE optimized geometry to better estimate the bandgap energy. A norm-conserved pseudopotential was utilized in all property calculations to replace core electrons in each atomic species.^36^ The following pseudopotentials were employed: P_00PBE_OP.recpot (for P atom), S_00PBE_OP.recpot (for S atom), and Nb_00.recpot (for Nb atom). These pseudopotentials are produced through a kinetic energy optimization scheme developed by Lin et al.^36^ and Lee.^37^ DFPT linear response calculations^38,39^ were used to calculate the vibrational properties, such as infrared (IR) and Raman intensities and their active modes.

A Monkhorst–Pack grid^40^ 6 × 6 × 1 is utilized to calculate all integrals in reciprocal space, which is enough to provide a well-converged electronic structure, taking into account the following electronic valence configuration: Nb-4*d* ^4^5*s* ^1^, P-3*s* ^2^3*p* ^3^, and S-3*s* ^2^3*p* ^4^.

For convergence criteria, the following parameters for self-consistent steps were used: a total energy change under 1.0 × 10^–5^ eV/atom, forces on all atoms below 0.03 eV/Å, pressure less than 0.05 GPa, and maximum allowed displacements of atomic positions of no more than 1.0 × 10^–3^ Å,^18,41^ through the Broyden–Fletcher–Goldfarb–Shanno minimizer.^42^ The cutoff energy of the plane waves was set to 650 eV in all property calculations.

The formation energy was calculated using DMol^3^ software.^43,44^ To calculate the formation energy, the following convergence tolerance parameters were used: a total energy change of less than 1.0 × 10^–5^ Ha, ta maximum force of 0.002 Ha/Å, and maximum allowed displacements of atomic positions no greater than 0.005 Å. The *k*-point set utilized was 4 × 4 × 4 for the Nb, P, and S structure references and also for NbPS_3_. The DNP basis set with the DSPP^45^ norm-coserved pseudopotential, which introduces some degree of relativistic correction into the core, was employed for the formation energy calculations.

Ab initio molecular dynamics (AIMD) simulations were carried out using the QuantumATK W-2024.09 package.^46–49^ Thermal stability was evaluated considering spin-polarized GGA-PBE quantum dynamics simulations at 300 and 600 K within the NVE ensemble, utilizing the Verlet velocity method,^50^ following the same scheme as previous work.^18^ The Maxwell–Boltzmann distribution was used to set the initial velocities. The medium basis set and the PseudoDojo norm-conserving pseudopotentials^48^ were used in the simulations, with a 4 × 4 × 4 *k*-point mesh, a density cutoff of 45 Ha, a total simulation duration of 5 ps, and a time step of 1 fs.

## Results and Discussion

### Geometry Optimization

The primitive cell of the monolayer NbPS_3_ is shown in Figure 1, and their lattice parameters for the cell (*a = b*) are shown in Table 1, obtained from the GGA-PBE approach, corresponding to the symmetry group *C2/m*. The internal atomic coordinates for NbPS_3_ are presented in Supporting Information Table S3. Brillouin zone of the NbPS_3_ monolayer, showing high-symmetry points, is depicted in Figure 2. To ensure a monolayer structure, the lattice parameter c was chosen to be larger than 20 Å, as is suggested in ref 51.

**Figure 1:**
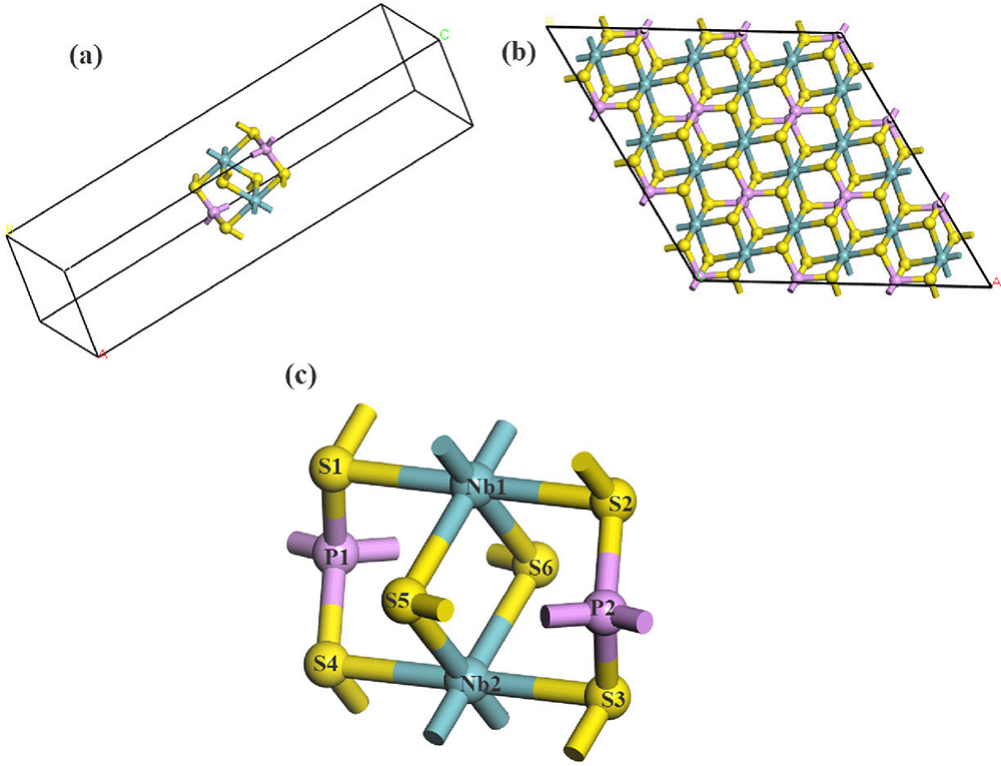
(a) NbPS_3_ primitive cell, (b) NbPS_3_ cell replicated in the *a* and *b* directions, and (c) primitive cell highlighting atom positions. The structure consists of Nb (blue ball), P (purple ball), and S (yellow ball).

**Figure 2:**
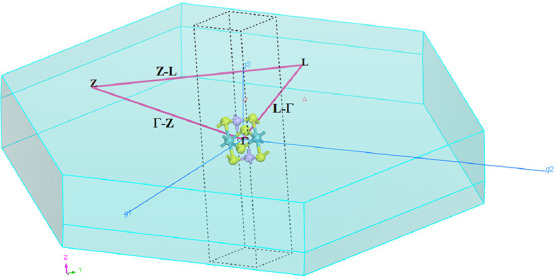
Brillouin zone of the NbPS_3_ monolayer, showing high-symmetry points (Γ-Z-L) and the primitive vectors ( ${\vec{g}}_{1}$ , ${\vec{g}}_{2}$ , and ${\vec{g}}_{3}$ ) of their reciprocal lattices. The structure consists of Nb (blue ball), P (purple ball), and S (yellow ball).

**Table 1:** Lattice Constants of the NbPS_3_ Monolayer According to the GGA-PBE Approach^a^

monolayer	*a*	*b*	*c*	α	β	γ
NbPS_3_	5.869	5.869	28.698	97.843	97.843	120.058

a Lengths (*a,b,c*) are in Å, and angles (α, β, γ) are in degrees (°).

The optimized lattice parameters *a* and *b* (Table 1) are in good agreement with the experimental values of the corresponding bulk structures of FePS_3_, MnPS_3_, CdPS_3_, CoPS_3_, and NiPS_3_,^52^ indicating a reliable description of the NbPS_3_ geometry.

All calculated bond length results were obtained by considering the GGA-PBE functional. The calculated Nb–S bond length is 2.587 Å for the NbPS_3_ monolayer (see Supporting Information Table S1). For comparison, the experimental Nb–S bond distance of NbS_3_ varies from 2.488 to 2.670 Å,^53^ which is close to the Nb–S bond lengths for NbPS_3_. The P–S and P–P bond lengths for the NbPS_3_ monolayer are 2.040 and 2.233 Å, respectively. The experimental values of the P–S bond lengths for the molecular crystal structures P_4_S_10_ and P_4_S_7_ vary from 1.93 to 2.12 Å,^54^ which is consistent with P–S (2.040 Å) bond lengths for the NbPS_3_ monolayer. In black phosphorus, the reported bond length between the two nearest P atoms is 2.224 Å,^55–57^ which is close to the length of the P–P bond (2.233 Å) for NbPS_3_. The Nb–S bond length (2.587 Å) in the NbPS_3_ monolayer (Supporting Information Table S1) is close to the experimental average values of M–S reported for FePS_3_ (2.548 Å) and MnPS_3_ (2.625 Å), indicating similar metal–sulfur bonding characteristics within the MPX_3_ family.^52^ The calculated P–S bond lengths (2.040 Å) also agree with the experimental range of 2.029–2.034 Å for other MPX_3_ materials. The P–P bond of NbPS_3_ (2.233 Å) is also compared to bulk MPX_3_ materials (2.148–2.222 Å).^52^

### Cohesive Energy and Formation Energy

The stability and integrity of a nanomaterial are indicated by the cohesive energy, which is a measurement of how firmly atoms are bound within a crystal structure.^18,58^ It is the energy needed to decompose the solid into independent atoms, each at 0 K.^59^ The formal definition of cohesive energy, *E*_coh_, is the difference between the sum of the energy of each atom present in NbPS_3_ and the total energy of the respective monolayer divided by the number of atoms in the unit cell. For NbPS_3_, the cohesive energy is as follows:^60–64^

$$E_{\mathrm{coh}}=\frac{2E_{\mathrm{Nb}}+6E_{S}+2E_{P}-E_{{\mathrm{NbPS}}_{3}}^{\mathrm{Tot}}}{10}$$ 1

where *E*_Nb_, *E*_S_, *E*_P_, and *E*_NbPS_3__^Tot^ are the energies of Nb, S, and P atoms, and the total energy of the NbPS_3_ monolayer, respectively. The *E*_Nb_, *E*_S_, *E*_P_, and *E*_NbPS_3__^Tot^ values are −133.120, −273.664, −173.937, and −2314.309 eV, respectively. Thus, the cohesive energy (*E*_coh_) obtained for NbPS_3_ is 5.821 eV/atom. This result shows that the monolayer studied has a high cohesive energy and consequently, high stability. The cohesive energy obtained for NbPS_3_ is higher, e.g., than phosphorene (3.48 eV/atom) and silicene (3.96 eV/atom) monolayers.^18,65^

The formation energy (*E*_form_) was also calculated for NbPS_3_ using the following equation:^63,66–68^

$$E_{\mathrm{form}}=E_{{\mathrm{NbPS}}_{3}}-\sum_{i}n_{i}\mu_{i}$$ 2

where *n*_*i*_ is the number of atoms of each element (Nb, P, and S) in the NbPS_3_ monolayer, μ_*i*_ is the chemical potential, and *E*_NbPS_3__ is the total energy of the NbPS_3_ monolayer. DMol^3^ software was used to calculate the formation energy of the NbPS_3_ monolayer. To estimate the formation energy, the total energies of the most stable periodic structures for Nb, P, and S were evaluated, and the associated atomic energies were taken as their chemical potentials.^18^ For Nb, the bcc phase corresponding to the space group *Im3̅m* (2 atoms per unit cell) was considered. For P, the reference was black phosphorus, and the space group *Cmca* (8 atoms per unit cell) was considered. For S, the orthorhombic space group *Fddd* (128 atoms per unit cell) was employed. The calculated formation energy for NbPS_3_ is −5.344 eV. For comparison, the formation energy of the MoS_2_ monolayer is −2.70 eV in GGA-PBE.^66^

### Electronic Properties

The band structure and partial density of states (PDOS) of the NbPS_3_ monolayer are shown in Figure 3. The Fermi level energy (dashed black line) at 0.0 eV was defined to correspond to the valence band maximum. It is possible to note the semiconductor characteristic of the NbPS_3_ monolayer with a direct bandgap in Γ of 0.630 eV when considering the GGA-PBE functional. This result shows that due to the direct bandgap feature of the NbPS_3_ monolayer, this material could be suitable, for example, for solar cell applications. When considering the HSE06 functional, the direct bandgap remains at Γ of 0.872 eV, as observed in Figure 3. These results for NbPS_3_ compare well with other phosphorus trichalcogenides whose bandgap varies between 1.2 and 3.5 eV.^21–24^ Furthermore, the experimental optical bandgap energy of the 30 nm thick FePS_3_ nanosheet was estimated to be 1.23 eV.^69^

**Figure 3:**
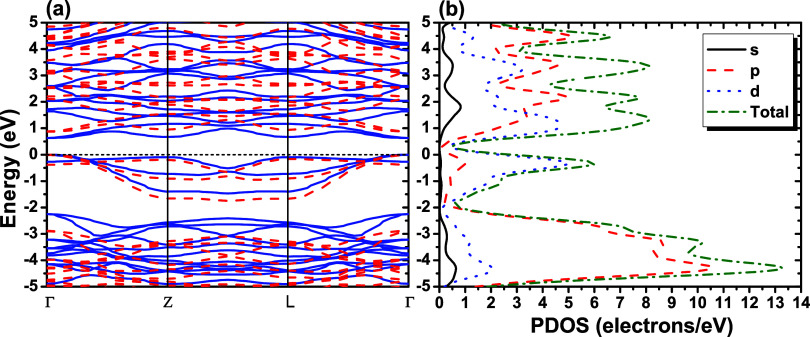
(a) Kohn–Sham band structures for NbPS_3_ using GGA-PBE (solid blue lines) and HSE06 (dashed red lines). (b) Partial density of states (PDOS) was calculated using GGA-PBE. The solid black line, dashed red line, dotted blue line, and dashed-dotted olive line represent the *s*, *p*, and *d* orbitals and total DOS, respectively.

The bandgap value allows one to estimate the electrical power conversion efficiency (PCE) of the NbPS_3_ monolayer for potential applications in solar cells. Rühle^70^ presents the theoretical limit of the PCE and other parameters for single-junction photovoltaic solar cells as a function of the bandgap. Considering bandgap on the HSE06 functional of 0.872 eV and comparing with Table 1 in ref 70, an estimated PCE (η) for NbPS_3_ can be found between 25.13 and 28.64%. These values should be regarded only as theoretical upper bounds, serving to place the bandgap of NbPS_3_ in the broader context of photovoltaic materials.

In Figure 3b, the partial density of states (PDOS) is shown, allowing visualization of each atomic orbital contribution within the energy range of interest. For the NbPS_3_ monolayer, the atomic orbitals are as follows: Nb-4*s* ^2^, 4*p* ^6^, 4*d* ^4^, 5*s* ^1^, P-3*s* ^2^, 3*p* ^3^ and S-3*s* ^2^, 3*p* ^4^. In the range of −5.0 to 0 eV, the main contributions in the energy bands are related to Nb-*d* ^4^, P-*p* ^3^, and S-*p* ^4^ (see Supporting Information Figures S1–S3). Above the Fermi level, in the range of 1.0–4.0 eV, the main contributions in the energy bands are related to the Nb-*d* ^4^ orbital.

A comparison of the PBE and PBE+SOC electronic band structures, shown in Figure 4, indicates that the inclusion of SOC induces only minor changes near the Fermi level, leading to a negligible effect on the electronic bandgap of NbPS_3_. The PBE+SOC calculations predict direct bandgaps of 0.621 eV, while the calculations without SOC predict direct bandgaps of 0.630 eV.

**Figure 4:**
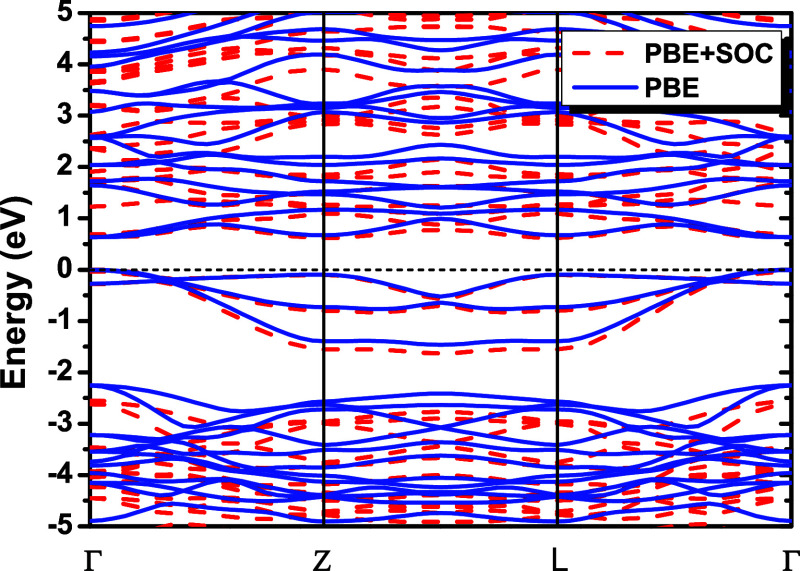
Kohn–Sham band structures for NbPS_3_ using GGA-PBE (solid blue lines) and PBE+SOC (dashed red lines).

### Magnetic Properties

In general, spin-polarized calculations within DFT are an exceptional tool to investigate magnetism in solid-state materials.^71^ The asymmetric band structure and DOS (Figure 5) confirm the presence of a magnetic moment in the NbPS_3_ structure. Furthermore, in the spin-polarization band structure, Figure 5, a metallic behavior can be observed in the spin-down valence band (dashed red lines). Thus, the GGA-PBE functional reveals that NbPS_3_ has a half-metallic ground state. Half-metallicity is a sought-after characteristic in spintronic devices,^72^ making NbPS_3_ a promising candidate for this application.

**Figure 5:**
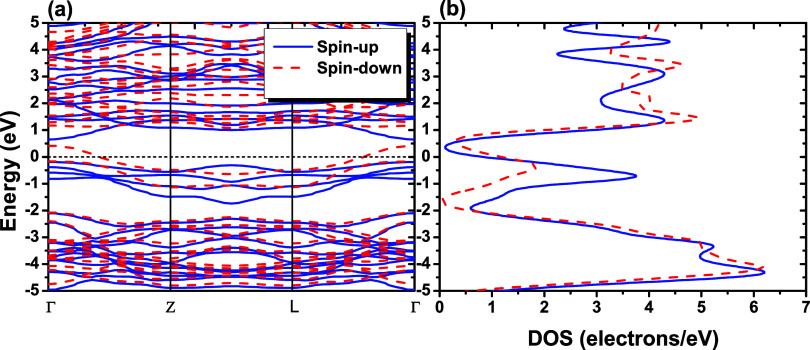
(a) Kohn–Sham spin-polarized electronic band structure and (b) total density of states (DOS) for the NbPS_3_ monolayer, using the GGA-PBE exchange–correlation functional, with blue lines representing the spin-up state and dashed red lines representing the spin-down state. The Fermi level is set as 0.0 eV.

Supporting Information Table S2 shows the magnetic spin moment for each atom, considering Mulliken^73^ and Hirshfeld^74^ population analyses. The sum results in a total unbalanced spin density of around 2.0 μB for the NbPS_3_ cell. Furthermore, the difference of 2 × Integrated |spin density| (2.29 μB) and 2 × Integrated spin density (2.00 μB) is finite, indicating uncompensated spin polarization and ferromagnetic behavior. However, this criterion alone does not guarantee that the ground state is ferromagnetic. According to Torun et al.,^72^ ferromagnetically ordered magnetic moments are a crucial requirement for spintronic devices. NbPS_3_ may have potential applications in spintronics if its magnetic properties are further verified.

It is reported in the literature that MPS_3_ materials with M = V, Mn, Fe, Co, and Ni are typically antiferromagnetic Mott insulators with variable semiconducting energy gaps and magnetic structures.^75^ Furthermore, MnPS_3_ and MnPSe_3_ monolayers showed antiferromagnetic Néel order.^76^ DFT calculations predicted that the magnetic ground state of intrinsic FePS_3_ can be converted from antiferromagnetic to ferromagnetic by adsorbing H atoms.^77^ In addition, theoretical studies in FePS_3_ show that in higher fields, a transition is observed from the antiferromagnetic (AFM) ground state to the half-metallic ferromagnetic (FM) state.^78^ Moreover, the experiment demonstrated that the strong out-of-plane ferromagnetism emerges from antiferromagnetic FePS_3_ single crystals after PyH^+^ intercalation.^79^ It was also observed that both electron and hole doping induce a magnetic transition from AFM to FM half-metal in two-dimensional MnPSe_3_.^80^ All of this evidence reinforces the fact that the MPX_3_ family is magnetically versatile. The fact that NbPS_3_ shows indications of ferromagnetic characteristics, in contrast to the antiferromagnetic properties of other transition-metal phosphorus trichalcogenides, could lead to the development of new applications for this class of materials. However, a deeper investigation is still required to confirm ferromagnetism as the true ground state.

### Optical Properties

Figure 6 shows the calculated optical absorption in nanometers (nm) for the NbPS_3_ structure, considering the incident light polarized with respect to the polycrystalline sample (Poly) and in directions [100], [010], [001], [110], [101], [011], and [111] employing the GGA-PBE functional. It is evident in Figure 6 that the optical absorption of NbPS_3_ depends on the directions of the incident electromagnetic radiation, showing anisotropy. For all directions, NbPS_3_ shows higher absorption in the ultraviolet region (∼100–400 nm) of the electromagnetic spectrum. This result suggests that NbPS_3_ could be used as a potential UV (ultraviolet) detector or as a UV filter. The most intense peak in the UV region (around 135 nm) corresponds to directions [001], [101], [011], and [111]. Also, in the UV region around 210 nm, there is an intense absorption peak corresponding to directions [100], [010], and [110]. The NbPS_3_ monolayer exhibits a pronounced optical response within the visible range. Between 640 and 660 nm, corresponding to the red color region, the directions Poly, [100], [110], and [010] represent an intense peak of absorption. The intense absorption peak in UV is probably due to interband transitions between deeper valence bands (predominantly p states for P and S atoms) and Nb d conduction states, as shown in the Supporting Information PDOS figures. A similar discussion could explain an intense peak in the red region. Thus, the red/visible peak corresponds to lower-energy interband transitions involving the topmost valence bands (p states for S and P) and higher-lying Nb d conduction bands. Optical absorption in the visible region of the spectrum for monolayers is important for applications in the optoelectronics and photonics fields. The reasons for this importance are related to applications such as photodetectors, sensors, and solar cells. The absorption in the visible region of NbPS_3_ makes it a potential 2D material for applications in photodetectors and sensors that could be used, e.g., for environmental monitoring and biomedical imaging. Photoluminescence observed experimentally in a multilayered 2D MnPS_3_ ^81^ supports the presence of optical activity in MPX_3_ compounds. In addition, photoluminescence was measured in CdPS_3_ intercalated with Ce and Eu ions,^82^ reinforcing the potential of the NbPS_3_ monolayer for visible-range optoelectronic applications. Furthermore, NbPS_3_ could be employed in solar cell applications for two main reasons: the direct bandgap feature of NbPS_3_ and its absorption in the visible region, which is critical for efficient solar energy conversion, because visible light accounts for ∼50% of solar radiation reaching Earth’s surface.^83^

**Figure 6:**
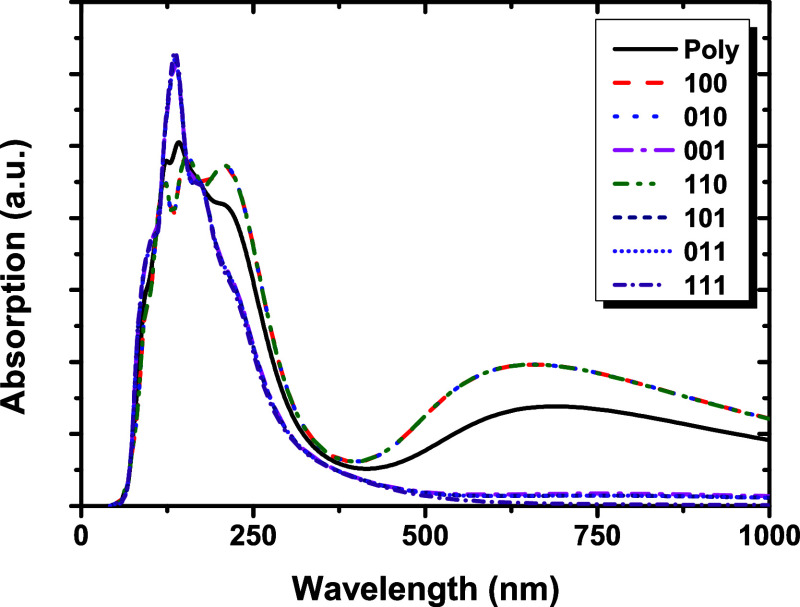
Optical absorption spectra for NbPS_3_ using the GGA-PBE exchange–correlation functional when the incident radiation is polarized along the crystalline planes [100, 010, 001, 110, 101, 011, and 111] and for the polycrystalline sample (Poly).

As shown in Figure 7, the real and imaginary dielectric function (ε) as a function of the frequency (eV) for the NbPS_3_ monolayer. The complex dielectric function is directly related to the electronic band structure^84^ and describes the dispersive properties of materials.^85^ The real (ε_1_) and imaginary (ε_2_) components of the dielectric function provide significant information about the loss of light absorption due to polarization and the retardation of the velocity of light.^85^ The dielectric function of the NbPS_3_ monolayer is highly anisotropic in the low energy range (0 to 2.5 eV) and the region between 5 and 10 eV and tends to be isotropic after 15 eV.

**Figure 7:**
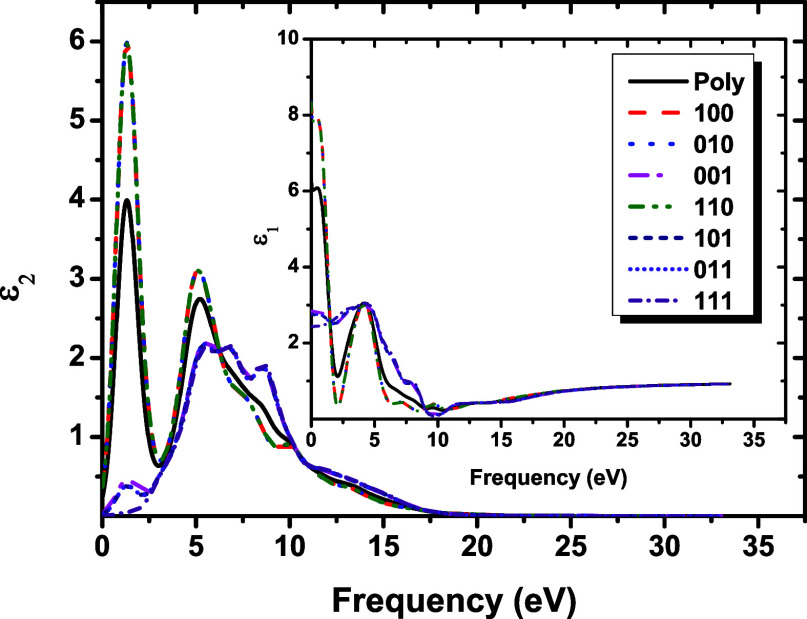
Dielectric function ε (imaginary part ε_2_ in the external picture and the inset show the real part ε_1_) as a function of the energy (eV) for NbPS_3_ considering the incident light polarized along distinct crystalline planes and for the light incident on a polycrystalline sample (Poly) using the GGA-PBE exchange–correlation functional.

The imaginary dielectric constant calculated is higher in directions Poly, [100], [010], and [110] (around 1.3 and 5.0 eV) and in [001], [101], [011], and [111] (around 5.3, 6.7, and 8.6 eV). In the real part of the dielectric function, the most prominent peaks occur in the region up to 0.0 eV in the following directions: Poly, [100], [010], and [110], and in the region around 4.0 eV, all directions present pronounced peaks. This is due to electronic transitions that involve the valence states P-*p* ^3^ and S-*p* ^4^ characters to the Nb-*d* ^4^ conduction states (see Supporting Information Figures S1–S3).

### Thermodynamic Properties

Figure 8a displays the thermodynamic potential curves, such as enthalpy, *T* × entropy, and free energy, as a function of temperature (*T*), of the two-dimensional niobium phosphorus trichalcogenide (NbPS_3_). Thermodynamic potentials were calculated using NbPS_3_ vibrational frequencies that were obtained from density functional perturbation theory (DFPT),^38,39^ as implemented in the CASTEP code. It is possible to note that the enthalpy curve (black solid line) exhibits a nearly linear behavior between 0 and 1000 K. The free energy (red dashed line) of the NbPS_3_ monolayer decreases slightly to about 600 K and then shows a linear temperature dependence. Furthermore, the free energy remains negative for all temperatures between 0 and 1000 K.

**Figure 8:**
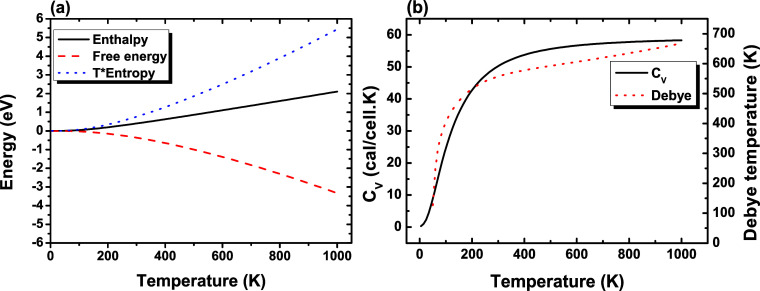
Thermodynamic potentials for NbPS_3_: (a) enthalpy (solid black line), free energy (dashed red line), and *T* × entropy (dotted blue line) as functions of temperature, obtained using the GGA-PBE exchange–correlation functional. (b) Constant volume heat capacity *C*_V_ (solid black line), as a function of the temperature (in K) for NbPS_3_. The red dotted line, using the right-hand side scale, depicts the temperature dependence of the Debye temperature.

Figure 8b shows the constant volume heat capacity (C_V_) as a function of temperature (solid black line) and, on the right-hand side scale, the temperature dependence of the Debye temperature (red dotted line), both calculated using the GGA-PBE functional. Heat capacity is the energy, in the form of heat, that any material must absorb for the temperature to increase by 1 K.^86^ From Figure 8b, the heat capacity increases rapidly as the temperature increases from 0 to 600 K, following the Debye *T* ^3^ law at low temperatures (0–200 K) before saturating at the Dulong–Petit limit in the high-temperature regime. The *C*_V_ increases with increasing temperature because heating leads to more phonons participating in it.^18,86^ The Debye temperature is a fundamental physical parameter that separates the low- and high-temperature vibrational behavior of a solid,^87^ providing information on how rigid the lattice is, that is, the higher the Debye temperature, the stronger the atomic binding and, consequently, the greater their thermal vibration.^88^ The Debye temperature in Figure 8b (red dotted line) increases with the temperature because it is estimated from the heat capacity curve. This behavior shows how more phonon modes become thermally activated and contribute to the lattice vibrations as the temperature increases. The temperature dependence of the Debye temperature curve behavior of NbPS_3_ is similar to that of other monolayers.^88,89^

Lastly, these findings can be utilized to forecast the structure’s stability for various two-dimensional metal phosphorus trichalcogenides or to compare the results with those of experimental data.

### Phonon Dispersion

The phonon dispersion curves were calculated within DFPT, as implemented in CASTEP.^38,39^ The acoustic sum rule (ASR) was explicitly imposed during the postprocessing of the dynamic matrices, ensuring that the three acoustic branches vanish at the Γ point (residual frequencies <1.0 cm^–1^). For the out-of-plane acoustic (ZA) mode, the quadratic dispersion expected for 2D modes was analyzed by fitting frequencies close to Γ to the ω = *Aq* ^2^ law. The best fit resulted in *A* ≈ 1231.6 cm^–1^ Å^2^ with an intercept of 4.6 cm^–1^, confirming the quadratic behavior. The irreducible representation for the phonons at the Γ point is Γ_vib_ = 8*A*_g_ + 7*B*_g_ + 6*A*_u_ + 9*B*_u_. At Γ, the three zero-frequency modes are associated with the acoustic branches (one *A*_u_ and two *B*_u_). The phonon dispersion curve of the NbPS_3_ monolayer is presented in Figure 9. This figure shows the phonon dispersion for the NbPS_3_ monolayer, showing a frequency range of 0 to 600 cm^–1^. The vibrational frequencies were analyzed along the high symmetry directions (Γ-Z-L-Γ). The phonon dispersion curves in Figure 9 show no negative frequencies, suggesting that NbPS_3_ is dynamically stable. There are 30 vibrational modes, three of which are acoustic, appearing in the region from 0 to 125 cm^–1^. In the region from 100 to 350 cm^–1^, there are twenty-one contributions associated with the optical modes, and from 350 to 600 cm^–1^, there are six optical modes. These optical modes are associated with the IR- and Raman-active modes shown in Table 2.

**Figure 9:**
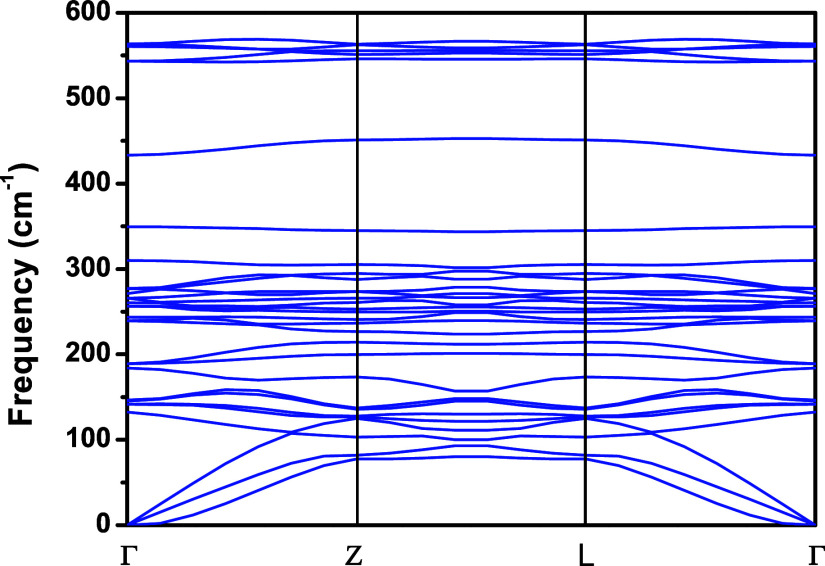
Phonon dispersion curves of NbPS_3_ in the frequency range from 0 to 600 cm^–1^, calculated by using the GGA-PBE functional.

**Table 2:** Computed Raman- and IR-Active Mode Frequencies of NbPS_3_

wavenumber (cm^–1^)	IR	Raman
132.24	N	Y
141.38	Y	N
141.53	Y	N
145.07	N	Y
146.46	N	Y
183.83	Y	N
188.99	N	Y
189.25	N	Y
239.15	N	Y
239.37	N	Y
243.65	Y	N
256.41	Y	N
256.49	Y	N
260.53	N	Y
265.71	Y	N
265.94	Y	N
271.31	N	Y
277.05	N	Y
277.25	N	Y
310.06	Y	N
349.37	N	Y
433.20	Y	N
543.35	N	Y
543.40	N	Y
560.61	N	Y
563.29	Y	N
563.44	Y	N

### Quantum Dynamics

The thermal stability of NbPS_3_ was examined through spin-polarized quantum dynamics using GGA-PBE. NbPS_3_ retained its structure at 300 and 600 K, with no chemical bond breaking and only minor deformations observed. In Figures 10 and 11, the fluctuations of potential energy (eV) × time (ps) dynamics graphics are depicted. To emphasize the small fluctuations, the mean potential energy was subtracted from each point. This representation indicates that the deviations remain small over time. By comparing Figures 10 and 11, it can be observed that the energy fluctuations were smaller in the dynamics performed at 300 K than in those at 600 K. However, both dynamics present small fluctuations, indicating that the simulation is reliable and that NbPS_3_ is thermally stable.

**Figure 10:**
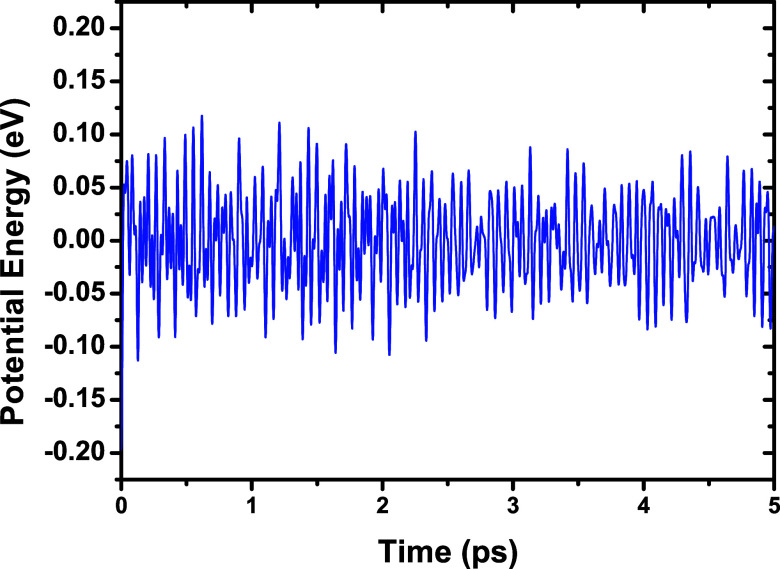
Potential energy fluctuations (eV) as a function of time (ps) for the NbPS_3_ monolayer in the AIMD simulation under the NVE ensemble at 300 K.

**Figure 11:**
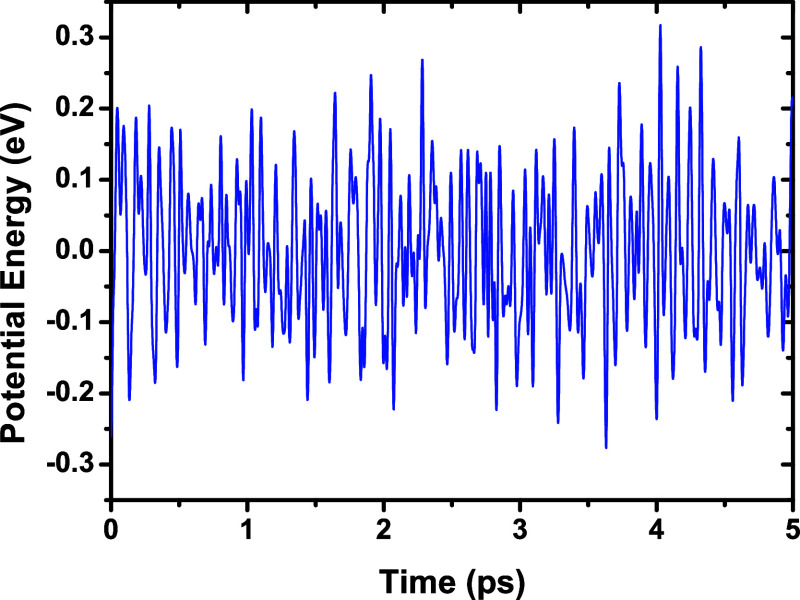
Potential energy fluctuations (eV) as a function of time (ps) for the NbPS_3_ monolayer in the AIMD simulation under the NVE ensemble at 600 K.

### Infrared and Raman Spectra

The DFPT linear response calculations,^38,39^ with the GGA-PBE functional, were used to calculate IR and Raman intensities and their active modes. In Figures 12 and 13, IR and Raman spectra are depicted for NbPS_3_. IR absorption intensities presented here refer to the dynamics matrix, known as Hessian, and to Born’s effective charges, also known as atomic polarizability tensors.^18,38,41^ The Raman spectrum, derived from the Raman shift produced by the inelastic scattering of monochromatic light,^41,90^ is employed to investigate vibrational, rotational, and low-frequency modes.^18^

**Figure 12:**
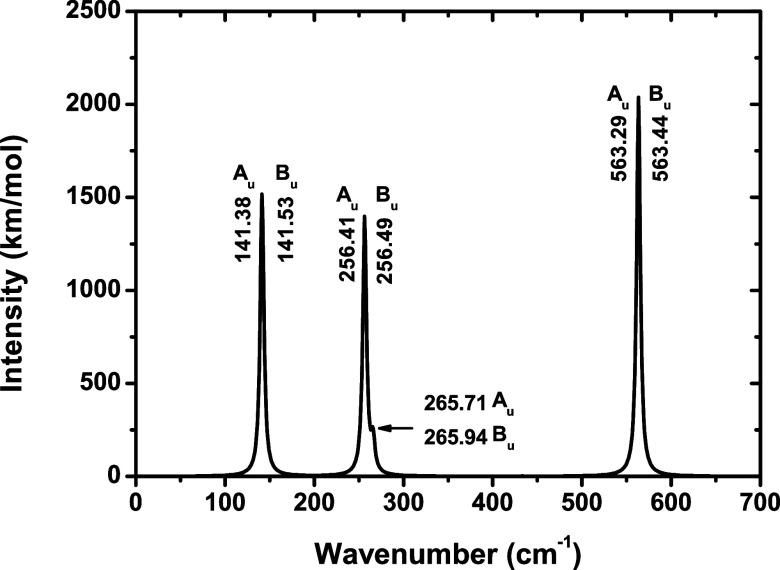
Infrared spectrum of NbPS_3_ in the 0–700 cm^–1^ range using the GGA-PBE exchange–correlation functional. The numbers denote the normal modes, with the irreducible representations also indicated.

**Figure 13:**
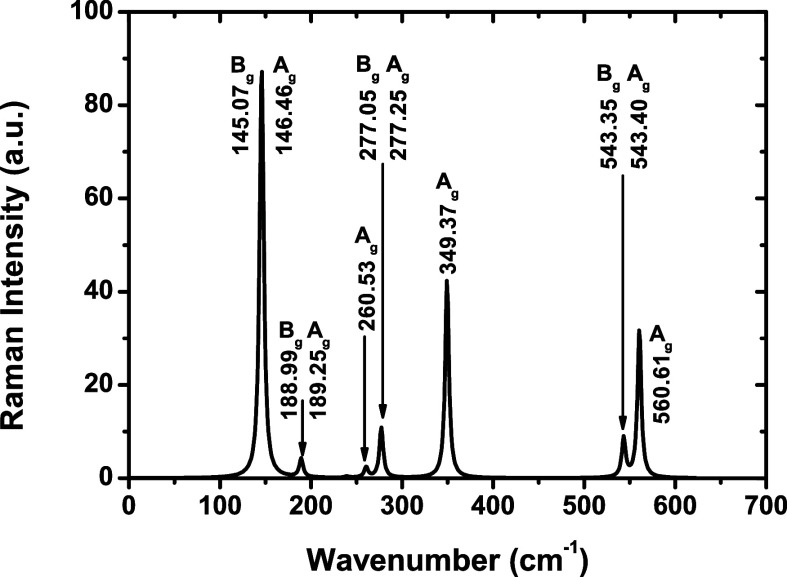
Raman scattering spectrum of NbPS_3_ in the 0–700 cm^–1^ range, using the GGA-PBE exchange–correlation functional. The numbers denote the normal modes with the irreducible representations also indicated.

All IR and Raman frequencies show positive frequencies, confirming that NbPS_3_ is at a local minimum. In Figures 12 and 13, the predicted normal modes are shown along with their corresponding irreducible representations, and in Table 2, the Raman- and IR-active modes are depicted. Figure 14 shows the atomic displacements of the most intense active IR modes. Although IR-active modes are calculated, including Born effective charges and the dielectric tensor, the nonanalytical correction responsible for the LO–TO splitting is applied; however, the resulting LO–TO separations are negligible (<0.2 cm^–1^, without showing any detectable separation) for this material.

**Figure 14:**
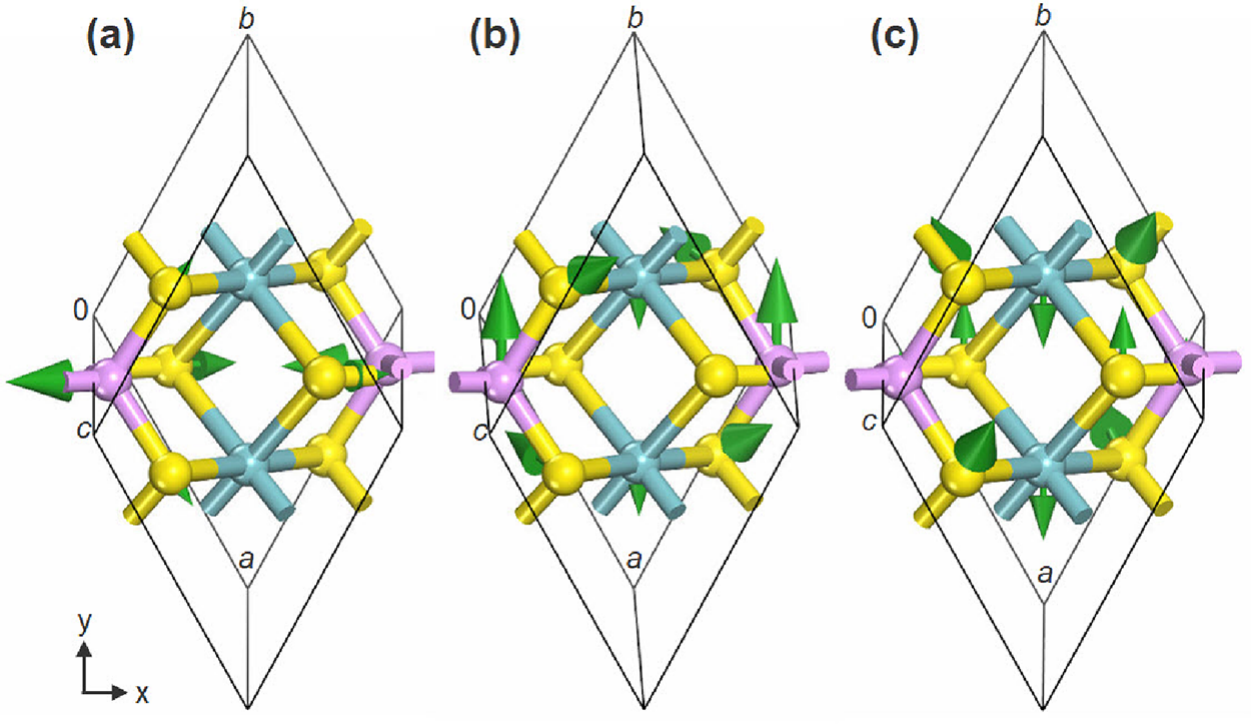
Vibration patterns of the most intense active IR modes observed in NbPS_3_: IR vibrational modes of S–P–S and S–Nb–S bonds at frequencies of (a) 563.44 cm^–1^ (*B*_u_ mode), (b) 141.38 cm^–1^ (*A*_u_ mode), and (c) 256.41 cm^–1^ (*A*_u_ mode). Green arrows highlight the vibrational mode tensors, and frequency and vibrational mode types are listed. The structures consist of Nb (blue ball), P (purple ball), and S (yellow ball).

The most intense IR peaks occur at 563.44 and 563.29 cm^–1^ [experimental frequencies for MPS_3_ compounds (bulk): 572 (MnPS_3_), 578 (FePS_3_), 571 (ZnPS_3_), and 575 cm^–1^ (NiPS_3_)^91^], which indicates that the P atoms move in the same direction parallel to the *x*- (see Figure 14a) and *y*-axis, associated with *B*_u_ and *A*_u_ modes, respectively, in the primitive cell and correspond to a S–P–S bending movement and an out-of-plane bending (wagging) movement of S–Nb–S. The second most intense peak is found in 141.38 and 141.53 cm^–1^ [152 (MnPS_3_), 151 (FePS_3_), 140 (ZnPS_3_), and 156 cm^–1^ (NiPS_3_)^91^], related to *A*_u_ and *B*_u_ modes, corresponding mainly to an out-of-plane bending (twisting) movement of the S–P–S bond and an out-of-plane bending (wagging) movement of the S–P–S bond, respectively (Figure 14b). Another more intense peak contains frequencies 256.41 and 256.49 cm^–1^ [255 (MnPS_3_), 258 (FePS_3_), 259 (ZnPS_3_), and 265 cm^–1^ (NiPS_3_)^91^], related to *A*_u_ and *B*_u_ modes, giving mainly an in-plane bending movement of the S–Nb–S bond and a rocking movement of the S–Nb–S bond, respectively (Figure 14c).

Figure 13 shows the Raman scattering spectrum profiles obtained from DFT for the NbPS_3_ monolayer in the range of 0–700 cm^–1^. Once out of that range, there are no absorption peaks. The most intense peaks comprise the modes *B*_g_ and *A*_g_ with frequencies 145.07 and 146.46 cm^–1^, respectively. Experimental frequencies for MPS_3_ compounds (bulk): 155 (MnPS_3_),^91^ 153.8 (MnPS_3_),^92^ 153 (FePS_3_),^91^ 156.9 (FePS_3_),^92^ 132 (ZnPS_3_),^91^ 176 (NiPS_3_),^91^ and 177.4 cm^–1^ (NiPS_3_).^92^ The second most intense peak for NbPS_3_ is found in 349.37 cm^–1^, related to an *A*_g_ mode [experimental values from ref 91: 385 (MnPS_3_), 378 (FePS_3_), 388 (ZnPS_3_), and 384 cm^–1^ (NiPS_3_); from ref 92: 383.2 (MnPS_3_), 380.9 (FePS_3_), and 384.8 cm^–1^ (NiPS_3_)]. The third most intense peak appears at 560.61 cm^–1^, assigned to an *A*_g_ mode [experimental values from ref 91: 568 (MnPS_3_), 573 (FePS_3_), 568 (ZnPS_3_), and 560 cm^–1^ (NiPS_3_); from ref 92: 567.5 (MnPS_3_), 582.9 (FePS_3_), and 589.2 cm^–1^ (NiPS_3_)]. In this interval (from 0 to 700 cm^–1^), other peaks are observed with their respective frequencies and modes, as shown in Figure 13 and Table 2.

## Conclusions

The present findings extend our understanding of the MPX_3_ family by introducing a novel NbPS_3_ monolayer as a structurally stable and semiconducting nanostructure. The ground-state properties of the NbPS_3_ monolayer characteristics, e.g., structure, electronic properties, optical absorption, phonon dispersion curves, IR, Raman, thermodynamic potentials, and heat capacity, were investigated using the well-established GGA-PBE functional. A HSE06 hybrid functional single-point energy calculation was performed starting from the optimized GGA-PBE geometry to better estimate the bandgap energy. The NbPS_3_ monolayer exhibits direct bandgaps (Γ → Γ) of 0.630 eV (GGA-PBE) and 0.872 eV (HSE06). Spin-polarized calculations showed potential ferromagnetic properties. The phonon dispersion of the NbPS_3_ monolayer does not present a negative frequency, indicating the possible stability of the system. Active IR and Raman modes were identified and assigned in the same wavenumber region, where high-intensity absorption bands are observed experimentally for MPS_3_ compounds. The absorption spectrum showed significant absorption in the UV region and showed anisotropy depending on the polarization of the incident radiation. Also, the NbPS_3_ monolayer presented robust absorption in the visible region, indicating that this trichalcogenide has potential for optoelectronic applications. The thermodynamic properties of the NbPS_3_ monolayer were studied, and it was shown that the free energy (*F*) assumes negative values for all temperatures considered. AIMD simulations showed the thermal stability of NbPS_3_ at temperatures of 300 and 600 K.

## Supplementary Material



## Notes

Structural data for the NbPS_3_ monolayer, including bond lengths (Table S1), atomic spin densities (Table S2), and internal atomic coordinates (Table S3); partial density of states (PDOS) plots for Nb, P, and S atoms, respectively (Figures S1–S3) (PDF)
