# Supplementary material for: Niobium Phosphorus Trichalcogenide (NbPS3): A Promising Monolayer Material for Magnetic and Optoelectronic Applications
Source: ACS Omega. 2025 Dec 19;11(1):944–54. doi: 10.1021/acsomega.5c07942 (PMC12809846; doi:10.1021/acsomega.5c07942)
Supplement: Supplementary file 1 [file ao5c07942_si_001.pdf]

SUPPORTING INFORMATION

Niobium phosphorus trichalcogenide ($NbPS_3$): A promising monolayer material for magnetic and optoelectronic applications

Leonardo de Souza Barbosa [a*], Willian Oliveira Santos [b], David Lima Azevedo [c], Edvan Moreira [a,d]

[a] *Postgraduate program in Aerospace Engineering, State University of Maranhão (UEMA), Cidade Universitária Paulo VI, 65055-310, São Luís, Maranhão, Brazil*

[b] *Postgraduate program in Physics, Federal University of Sergipe (UFS), Cidade Univ. Prof. José Aloísio de Campos, 49107-230, São Cristóvão, Sergipe, Brazil*

[c] *Institute of Physics, University of Brasilia (UnB), Campus Universitário Darcy Ribeiro - Asa Norte, 70919-970, Brasília, Distrito Federal, Brazil*

[d] *Department of Physics, State University of Maranhão (UEMA), Cidade Universitária Paulo VI, 65055-310, São Luís, Maranhão, Brazil*

* Corresponding author email address: leonardobarbosa@ppg.uema.br

Table S1 – Bond lengths of the $NbPS_3$ structure (distances in Å), considering the GGA-PBE functional.

Bond	Lenght (Å)
P2-S6	2.040
P1-S5	2.040
P2-S2	2.040
P1-S4	2.040
P1-S1	2.040
P1-S3	2.040
P1-P2	2.233
S3-Nb1	2.587
S1-Nb2	2.587
S4-Nb1	2.587
S2-Nb2	2.587
S2-Nb1	2.587
S4-Nb2	2.587
S3-Nb2	2.587
S1-Nb1	2.587
S6-Nb2	2.587
S5-Nb1	2.587
S5-Nb2	2.587
S6-Nb1	2.587

Table S2 – Atomic spin density for the $NbPS_3$ monolayer in μB units.

Atom	Mulliken	Hirshfeld
**P1**	0.021	0.03
**P2**	0.021	0.03
**S1**	-0.018	0.04
**S2**	-0.018	0.04
**S3**	-0.018	0.04
**S4**	-0.018	0.04
**S5**	-0.042	0.05
**S6**	-0.042	0.05
**Nb1**	1.056	0.83
**Nb2**	1.056	0.83
**Total (μB)**	2.00	1.98

Table S3: Internal atomic coordinates for $NbPS_3$. The coordinates (u, v, and w) are measured relative to the a, b, and c lattice parameters of the primitive cell, respectively.

**$NbPS_3$**	**Internal coordinates**		
**Element**	**u**	**v**	**w**
**P**	-0.053925	-0.053925	-0.540432
**P**	0.053925	0.053925	0.540432
**S**	-0.082155	-0.415913	-0.561534
**S**	0.415913	0.082155	0.561534
**S**	0.082155	0.415913	0.561534
**S**	-0.415913	- 0.082155	-0.561534
**S**	-0.748192	-0.748192	-0.561534
**S**	0.748192	0.748192	0.561534
**Nb**	-0.333244	-0.666756	-0.500000
**Nb**	0.333244	0.666756	0.500000

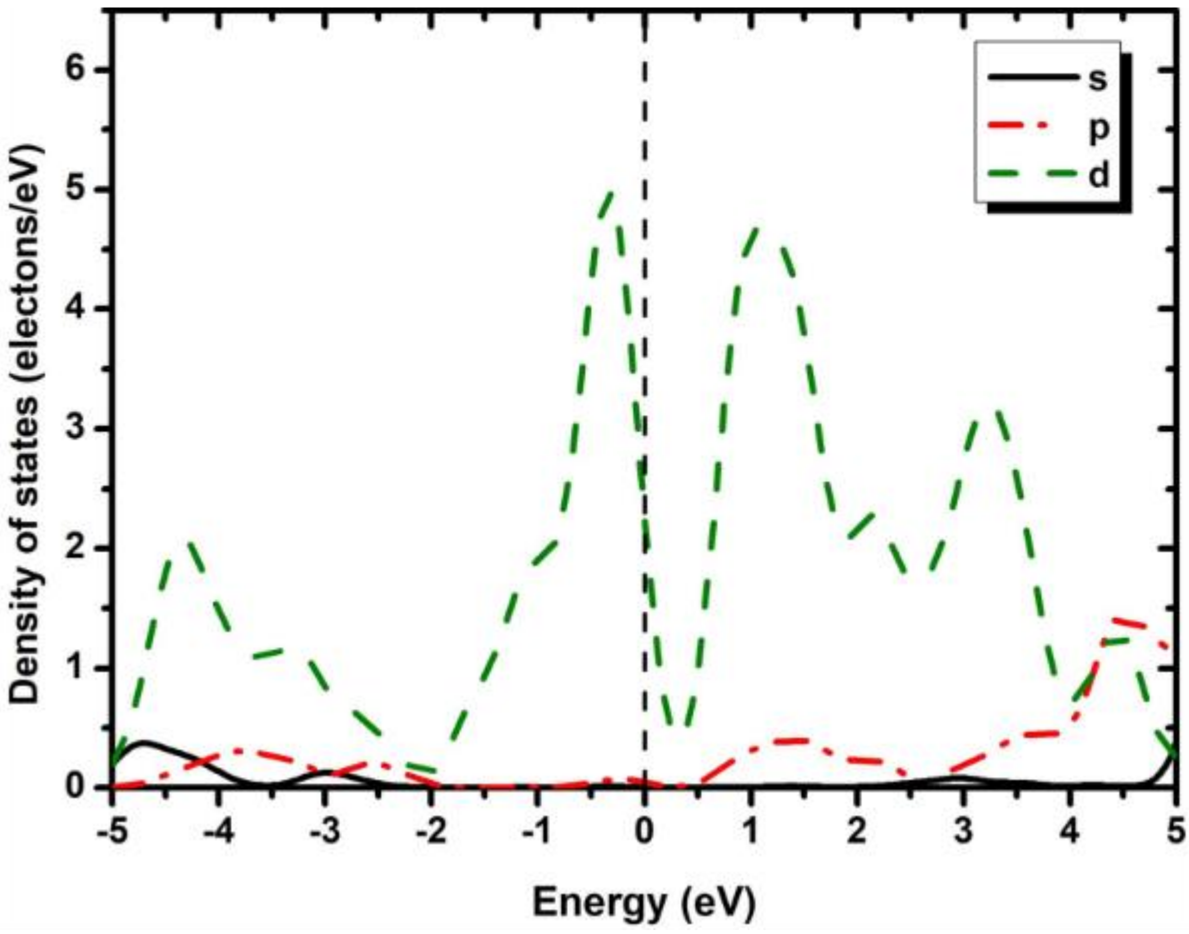

Figure S1 – Partial density of states (PDOS) of Nb atom calculated using GGA-PBE.

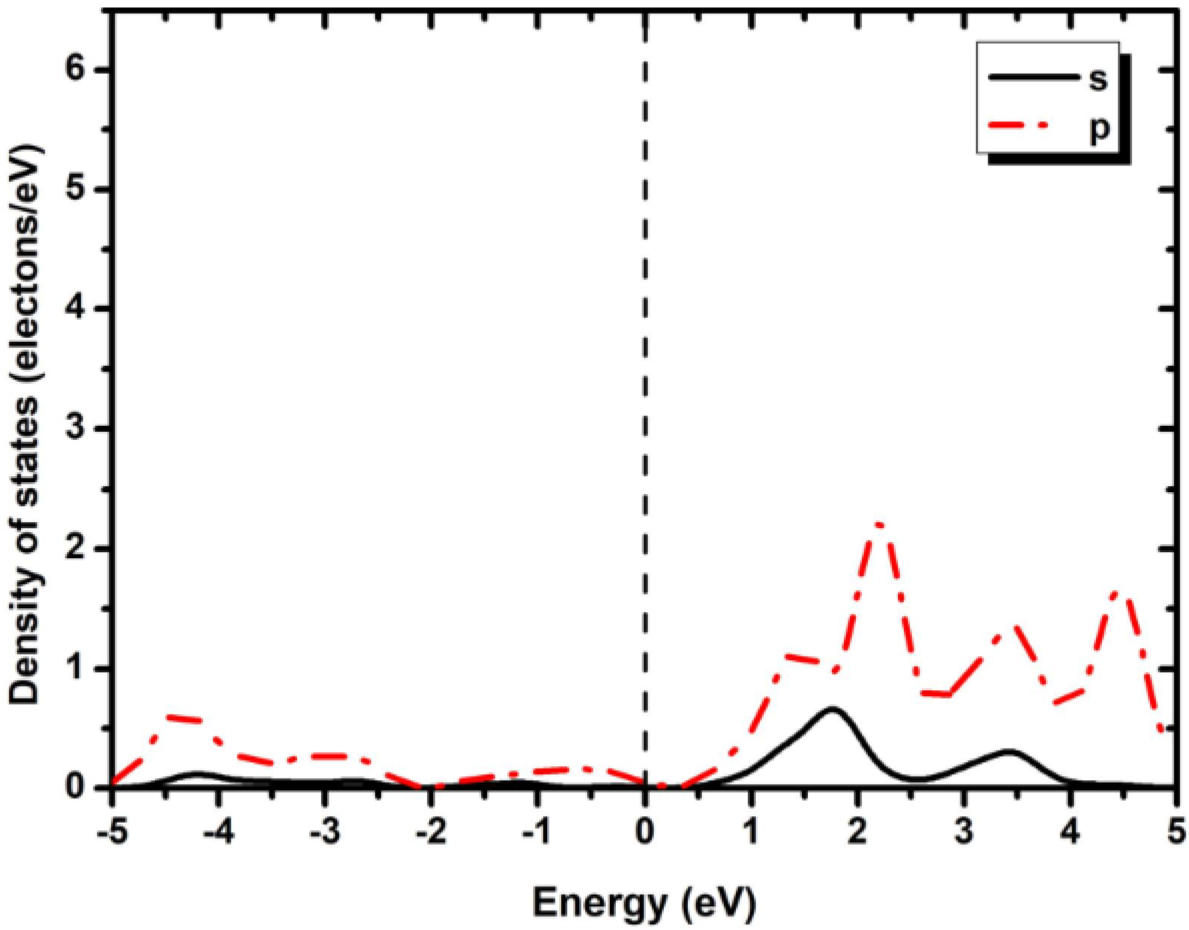

Figure S2 – Partial density of states (PDOS) of P atom calculated using GGA-PBE.

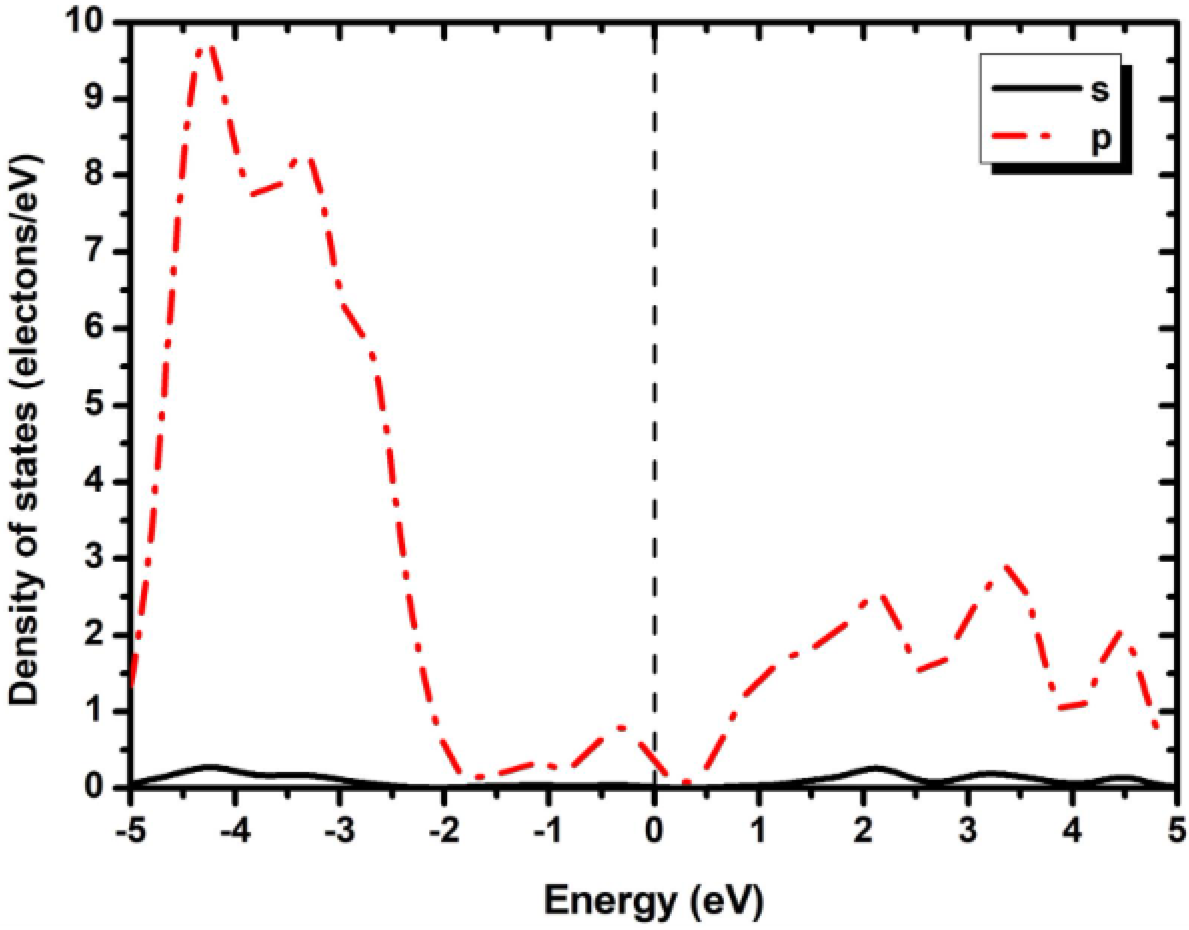

Figure S3 – Partial density of states (PDOS) of S atom calculated using GGA-PBE.
